# Protective effect of whey proteins against nonalcoholic fatty liver in rats

**DOI:** 10.1186/1476-511X-10-57

**Published:** 2011-04-13

**Authors:** Essam M Hamad, Soad H Taha, Abdel-Gawad I Abou Dawood, Mahmoud Z Sitohy, Mahmoud Abdel-Hamid

**Affiliations:** 1 Dairy Science Department, Faculty of Agriculture, Cairo University, Egypt; 2 Biochemistry Department, Faculty of Agriculture, Zagazig University, Zagazig, Egypt

## Abstract

**Background:**

Nonalcoholic fatty liver disease (NAFLD) is the hepatic manifestation of the metabolic syndrome and can vary from hepatic steatosis to end-stage liver disease. It is the most common liver disease and its prevalence is increasing worldwide. In the present study, the effect of whey proteins on some parameters of NAFLD was investigated.

**Results:**

Oral administration of the studied whey proteins products reduced the final body weight of rats. There was a significant reduction effect (*P *< 0.05) of the tested proteins on hepatic triglycerides, liver enzymes (ALT and AST), lipid peroxidation (malondialdehyde level) and serum glucose. Feeding on whey proteins caused an increase in the reduced glutathione. Hepatic content of reduced glutathione was not affected by any of the used whey proteins, but it showed an increasing tendency (*P *> 0.05). Liver histology showed an improvement of fatty infiltration in hepatocytes from whey protein groups and gives the histology of liver a normal appearance.

**Conclusions:**

The obtained results indicate a possible role for oral administration of whey proteins in the regulation of liver biochemistries in a rat's model of NAFLD. This regulatory effect of whey proteins was accompanied by an improvement in fatty infiltration in hepatocytes and a reduction of oxidative stress parameters.

## Background

Nonalcoholic fatty liver disease (NAFLD) is a wide spectrum of fatty liver changes ranging from hepatic steatosis (i.e. abnormal retention of lipids within a cell) to steatohepatitis (nonalcoholic steatohepatitis), which can progress to liver fibrosis and cirrhosis in the absence of alcohol consumption. NAFLD is rapidly becoming a worldwide public health problem. It is present in 10-24% of the world's population and its prevalence is even greater in obese individuals, ranging from 57.5 to 74.0% [1]. In Egypt, its prevalence in the morbidly obese population has been estimated as 22% for male and 48% for female [2]. Evidence from human studies and animal models have suggested that lipid accumulation in the liver (fatty liver) plays an important role in the pathogenesis of heart failure, obesity, diabetes and metabolic syndrome [3]. Treatment strategies for NAFLD aim to improve insulin sensitivity and modify metabolic risk factors, or protect the liver from oxidative stress. Therefore, the discovery of nutrients that ameliorate fatty liver is of interest.

Whey proteins (WP) represent a heterogeneous group of proteins (β-lactoglobulin, α-lactalbumin, serum albumin, immunoglobulins ...etc.). It has been suggested that WP has an antioxidant activity probably depending on the abundance of cysteine in WP or on the presence of glutamylcysteine groups which are in other food proteins. Therefore, WP may be considered as a possible therapeutical tool in oxidative stress correlated diseases [4]. An undenatured cysteine-rich whey protein isolate (WPI) has been proven to raise glutathione levels by supplying the precursors required for intracellular glutathione synthesis. This has been demonstrated in several glutathione-deficient patient groups including those with advanced human immunodeficiency virus (HIV) infection [5]. Whey protein concentrate (WPC), beta-lactoglobulin and alpha-lactalbumin improve the liver damage induced by CCl_4 _[4,6]. Oral administration of colostrum, which contains high amount of whey proteins, significantly decreased total cholesterol and triglycerides (TG) levels in both the men and women after 4 weeks [7]. Few studies used total whey proteins or WPI to study their effect on fatty liver [8,9]. Hence, this study aimed to investigate the protective effect of oral administration of different WP products against fatty liver in an experimental rat model.

## Results

### Growth parameters

Effect of oral administration of whey proteins on growth parameters of rats is presented in Table 1. Initial body weight of all rats was 138.8 ± 18.1 g. Non significant differences were found between body weight control rats and rats fed on high carbohydrate, fat free (HCFFD) diets (*P *> 0.05).

**Table 1:** Effect of oral administration of whey proteins products on final body weight, liver weight and hepatic index of rats fed the normal (control) and high carbohydrate, fat free (HCFFD) diets

	Final body weight (g)	Liver weight (g)	Hepatic Index
**Control**	180.32 ± 21.66 ^a^	6.27 ± 1.24^ab^	3.65 ± 0.73^a^
**HCFFD**	158.10 ± 25.77 ^ab^	6.53 ± 1.27^a^	3.96 ± 0.35^a^
**WPI**	126.48 ± 18.90^b^	4.22 ± 0.99^b^	3.32 ± 0.50^a^
**WPH**	131.70 ± 13.79^ab^	4.30 ± 1.62^ab^	3.25 ± 0.45^a^
**αLA**	128.76 ± 22.88^b^	4.67 ± 0.85^ab^	3.66 ± 0.60^a^
**βLG**	121.07 ± 8.13^b^	4.53 ± 0.33 ^ab^	3.74 ± 0.12^a^
**GMP**	155.19 ± 19.07^ab^	5.98 ± 1.15^ab^	3.85 ± 0.49^a^

Mean values with their standard deviation for five rats per group

WPI: whey protein isolate, WPH: whey protein hydrolysate, αLA: alpha lactalbumin, βLG: Beta lactoglobulin, GMP: glycomacropeptide.

Means in a column without common letter are significantly differ, *P *< 0.05 (Tukey's test).

Whey protein hydrolysate (WPH) and glycomacropeptide (GMP) rat groups also showed insignificant reduction in the final body weight of rats (*P *> 0.05). However, final body weight of rats decreased significantly when they were given whey protein isolate (WPI), α-lactalbumin or β-lactoglobulin.

Furthermore, liver weight of rats fed on control diet was similar to that of HCFFD rat group (*P *> 0.05). Compared with steatosis group (HCFFD), oral administration of WPI significantly (*P *< 0.05) decreased liver weight of rats, while the other whey proteins had non significant effect on the liver weight (P > 0.05). All the studied whey proteins had no significant effect on the hepatic index.

### Blood glucose and cholesterol and tissue triglycerides

Effect of oral administration of different whey proteins products on blood glucose and cholesterol and tissue triglycerides is presented in Table 2. Feeding rats on HCFFD was associated with a significant elevation of their blood glucose levels (76% elevation, *P *< 0.001) as compared with control diet group. It also appeared that oral administration of all the tested whey proteins lowered the blood glucose levels of rats, except for the α-Lactalbumin (αLA) group in which no significant difference was found as compared to the HCFFD group. Lowest levels of blood glucose were observed when rats were fed either WPI (86.69 ± 5.27 mg/dL) or GMP (95.02 ± 10.40 mg/dL) in which the levels of blood glucose were comparable with the control group (*P *> 0.05).

**Table 2:** Effect of oral administration of whey proteins products on serum glucose and total cholesterol and on liver triglycerides of rats fed the normal and high carbohydrate, fat free diets

	Blood glucose (mg/dL)	Total cholesterol (mg/dL)	TG (mg/g liver)
**Control**	86.69 ± 5.27^d^	71.92 ± 5.82^ab^	8.27 ± 1.08^b^
**HCFFD**	152.76 ± 20.81^a^	79.19 ± 9.85^a^	12.39 ± 2.32^a^
**WPI**	102.74 ± 10.96^cd^	69.29 ± 4.48^ab^	3.71 ± 1.64^c^
**WPH**	118.92 ± 10.61^bc^	63.74 ± 5.50^ab^	2.54 ± 0.31^c^
**αLA**	130.21 ± 16.95^ab^	51.72 ± 4.75^b^	3.07 ± 0.78^c^
**βLG**	115.06 ± 11.73^bc^	76.64 ± 7.47^ab^	2.58 ± 0.90^c^
**GMP**	95.02 ± 10.40^cd^	58.43 ± 6.55^ab^	2.59 ± 0.94^c^

Mean values with their standard deviation for five rats per group

WPI: whey protein isolate, WPH: whey protein hydrolysate, αLA: alpha lactalbumin, βLG: Beta lactoglobulin, GMP: glycomacropeptide, TG: triglyceride.

Means in a column without common letter significantly differ, *P *< 0.05 (Tukey's test).

The serum total cholesterol level of the HCFFD group tended to be higher (*P *> 0.05) than that of the control group. The lowest serum cholesterol level was obtained when rats were given the αLA (51.72 ± 4.75 mg/dL). It was also significantly different from that of HCFFD group (*P *< 0.05). The other whey proteins did not show any significant lowering effect on the serum total cholesterol of rats (Table 2).

The liver content of triglycerides (TG) is presented in Table 2. HCFFD group was characterized by its higher TG content (*P *< 0.001) as compared with that in the control diet. All the tested whey proteins showed a significant lowering effect on the liver content of TG compared with both the control and the HCFFD rat groups (*P *< 0.05). The lowest TG content was observed when rats were fed on WPH which was about 67% and 79% lower than that of the control and HCFFD groups, respectively.

### Serum alanine transaminase (ALT) and aspartate transaminase (AST)

**Figure 1:**
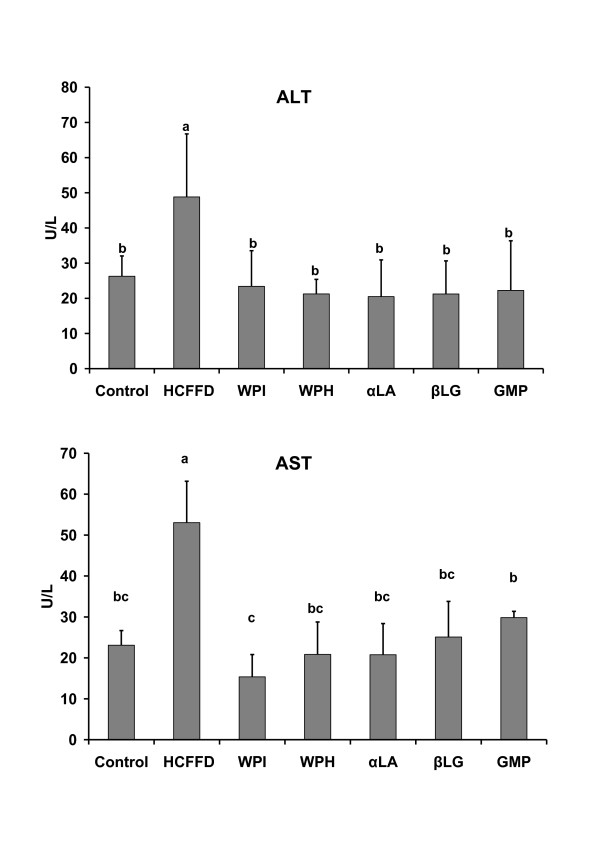
**Effect of oral administration of whey proteins products on serum alanine transaminase (ALT) and aspartate transaminase (AST) in rats fed the normal and high carbohydrate, fat free diets**.

Figure 1 illustrates the data of serum alanine transaminase (ALT) and aspartate transaminase (AST) as affected by the administration of whey proteins. The levels of ALT and AST increased significantly when rats were fed on HCFFD (*P *< 0.05) as compared with the control group. Oral administration of whey proteins under the present study lowered the serum levels of both enzymes to be in the range of the control group (*P *> 0.05). The lowest level of ALT was obtained when rats were fed on αLA (20.49 ± 10.0 U/L), while the lowest level of AST was observed when rats were given WPI (15.35 ± 5.46 U/L).

### Lipid peroxidation and oxidative stress

Data in Table 3 show the effect of whey proteins products on lipid peroxidation (malondialdehyde; MDA) and oxidative stress (reduced glutathione; GSH). It can be observed that MDA was significantly elevated in the HCFFD group as compared to the control group (*P *< 0.05). Feeding rats whey proteins was accompanied by lower MDA levels (*P *< 0.05), except for GMP group in which non significant differences were found between MDA levels and that of HCFFD group. On the contrary, GSH levels showed a tendency to be lower in the HCFFD group compared with the control (*P *> 0.05). Oral administration of WPI, WPH and βLG elevated the GSH levels significantly (*P *< 0.05, compared with HCFFD) while the rest of the whey proteins showed similar levels to the control.

**Table 3:** Effect of oral administration of whey proteins products on malondialdehyde and reduced glutathione in rats fed the normal and high carbohydrate, fat free diets

	Malondialdehyde (nmol/g liver)	Reduced Glutathione ( mg/g liver)
**Control**	220.33 ± 3.65^b^	91.58 ± 7.33^ab^
**HCFFD**	549.48 ± 7.99^a^	69.57 ± 8.37^b^
**WPI**	280.37 ± 1.42^b^	117.42 ± 5.50^a^
**WPH**	230.36 ± 7.45^b^	125.78 ± 3.56^a^
**αLA**	227.31 ± 6.12^b^	110.82 ± 4.92^ab^
**βLG**	229.99 ± 2.81^b^	115.27 ± 1.91^a^
**GMP**	365.56 ± 9.40^ab^	109.49 ± 2.70^ab^

Mean values with their standard deviation for five rats per group

WPI: whey protein isolate, WPH: whey protein hydrolysate, αLA: alpha lactalbumin, βLG: Beta lactoglobulin, GMP: glycomacropeptide.

Means in a column without common letter significantly differ, *P *< 0.05 (Tukey's test).

### Histological examination

**Figure 2:**
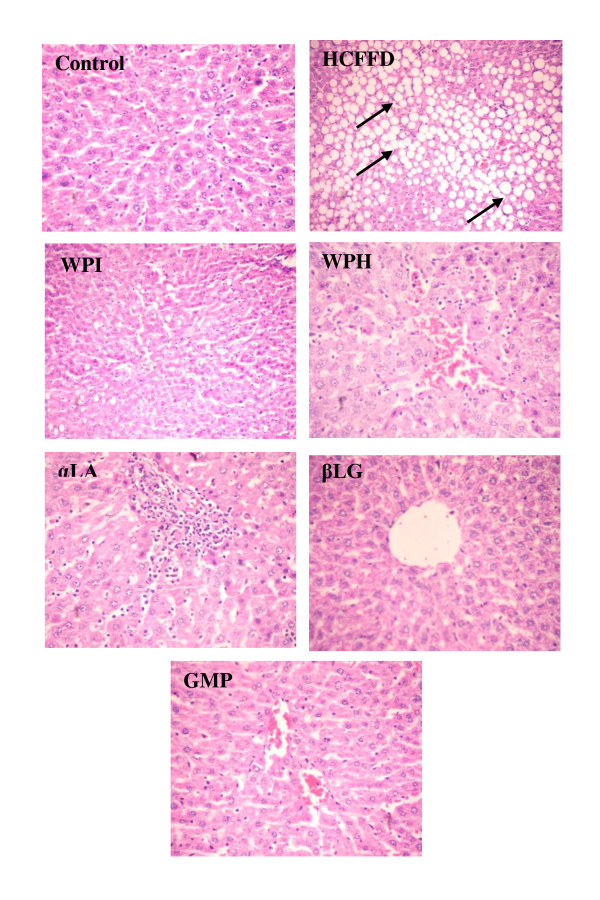
**Effect of oral administration of whey proteins products on the histological examination of livers from rats fed high carbohydrate, fat free diet for 4 weeks**. Control; basal diet group, WPI; whey protein isolate group, WPH; whey protein hydrolysate group, αLA; alpha lactalbumin group, βLG; beta lactoglobulin group and GMP; glycomacropeptide group. Arrows show numerous spherical vacuoles of fat droplets.

The histological examination of livers from the experimental rat groups is shown in Figure 2. It can be noticed that appearance of the liver from the control group was normal, and hepatocytes of this group exhibited normal morphology. On contrast, the appearance of the HCFFD group was looking like the fatty liver. Also, fatty infiltration and the fat vacuoles were found in more than 66% of hepatocytes macrovesicular vacuoles.

Oral administration of whey proteins products showed a positive effect on the histological examination of livers. The livers of all whey protein groups were improved and appeared with less fatty infiltration of hepatocytes as compared with HCFFD group (Figure 2).

## Discussion

Nonalcoholic fatty liver disease (NAFLD) occurs across all age groups and occurs in 14%-30% of the general population [10]. In this study, whey proteins products were assessed for their ability to improve the biochemical and histological features of disease activity in NAFLD induced by a high carbohydrate fat free diet in Wistar rats. Recently, few studies were concerned with the effect of whey proteins on the parameters of fatty liver [8,9]. Most of these studies focused on the use of total whey proteins instead of using their fractions individually. After 4 weeks of feeding high carbohydrate fat free diet, the histological appearance (Figure 2) of the positive control group (HCFFD) showed a typical appearance of fatty liver as reported by [11]. All tested whey proteins products led to an improvement in the results of histological examination of their livers (Figure 2). The hepatic triglycerides and the serum AST and ALT levels were improved in all whey protein groups. Further more, oral administration of all whey proteins products improved malondialdehyde levels.

In the current study, the growth parameters observation showed a reduction in the final body weight with oral administration of some whey proteins. These results are consistent with a variety of epidemiological trials whereas whey proteins or dairy products enhanced weight and fat mass loss in humans and animals [12,13]. However, not all weight loss studies were in agreement with this conclusion [14]. Therefore, the results of the current study confirm that oral administration of whey proteins may reduce weight gain in rats.

The key factor in nonalcoholic fatty liver disease is insulin resistance. Recent data clearly implicate hepatic insulin resistance as a responsible factor for the accumulation of free fatty acids as triglycerides in hepatocytes. Therefore, treatment strategies for fatty liver disease, at present, are primarily focused on weight loss and use of insulin sensitizing agents [15]. In this study, oral administration of all the tested whey proteins reduced serum glucose levels. At the same time, this beneficial effect was associated with a reduction in the hepatic triglycerides. Consequently, the histology of the livers of all whey protein groups appeared with less fatty infiltration in hepatocytes (Figure 2). This effect is similar to that observed by [8] who found that whey + calcium containing diet significantly decreased blood glucose, serum insulin, and hepatic triglycerides in lean and obese C57Bl/6J mice.

In addition, alanine transaminase (ALT) is an important enzyme found predominately in the liver. Significantly elevated activity of ALT in serum often suggests its leakage from damaged hepatic cells and reflects hepatocyte damage. Aspartate aminotransferase (AST) is similar to ALT in that it is another enzyme associated with liver. The ALT is a more specific indicator of liver inflammation than the AST, as the AST may also be elevated in diseases affecting other organs. For this reason, ALT is commonly used as a way of screening for liver problems. Elevated ALT levels correlated strongly with NAFLD [16,17]. In the present study, oral feeding of whey proteins lowered levels of ALT and AST in rats' model of fatty liver (Figure 1). This positive effect on ALT and AST might account for the improvement of the liver histology and less fatty infiltration in hepatocytes. These findings are in agreement with [9], who reported that a daily dose of 20 g whey protein isolate for 12 weeks given to fatty liver patients led to a significant reduction in ALT and AST.

Mitochondrial dysfunction is thought to play a central role in NLFLD as production of reactive oxygen species is increased and lead to inflammation, hepatocellular apoptosis, or in other words increased oxidative stress [9]. Hepatocyte and plasma glutathione (reduced form, GSH) decreased and glutathione disulfide (oxidized form, GSSG) increased in NAFLD patients [18]. As a consequence, strategies to increase glutathione concentrations to reduce oxidative stress might play an important role in pathogenesis of NAFLD. In the results of our study, a significant elevation in the GSH content was observed only when rats were fed on WPI, WPH or βLG (*P *< 0.05, compared with HCFFD). An undenatured cysteine-rich whey protein isolate has been proven to raise glutathione levels by supplying the precursors required for intracellular glutathione in several glutathione-deficient patient groups including those with advanced human immunodeficiency virus (HIV)-infection [19]. Also, total milk proteins did not decrease total liver GSH compared with the high sucrose diet group [20].

In addition, lipid peroxidation leads to the generation of by-products, such as malondialdehyde (MDA), which are involved in activation of the inflammatory response and consequently, cellular damage [19]. The results of high hepatic MDA levels in the steatosis group are in agreement with the results of [17]. Oral administration of whey proteins products was associated with lowering content of MDA in liver (Table 3). It is expected that the potential effect of whey proteins in preventing further accumulation of free radicals as well as oxidative stress could be due to their anti oxidant capacity and/or their anti-inflammatory effect. Previously, it was observed that whey protein led to an increase in the antioxidant enzymes and a decrease in the oxidative stress. Whey proteins also showed an anti-inflammatory effect in humans [21,22].

Oral administration of some whey proteins products (specially WPH and WPI) improved the fatty liver state in a rat model of NAFLD, which is in correlation with the results of histological examination of rats' liver and a reduction in hepatic triglycerides, liver enzymes levels (ALT and AST), serum glucose and lipid peroxidation (MDA). Further studies are needed to give further insight into the underlying mechanism.

## Materials and methods

This study was approved by the Committee of Scientific Ethics at Cairo University and according to its guidelines.

### Materials

Whey protein isolate (WPI), whey protein hydrolysate (WPH), α-lactalbumin (α-LA), β-lactoglobulin (β-LG) and glycomacropeptide (GMP) were kindly obtained from Davisco Food International (USA).

Male Wistar rats of average weight 138.8g were obtained from Animal House Lab., National Research Center, Cairo, Egypt.

Basal diet: a balanced basal diet (30% sucrose, 35% starch, 16% casein, 10% oil, 5% fiber and 4% minerals & vitamins) was obtained from Meladco Company, Egypt. High carbohydrate, fat-free diet (40% starch, 40% sucrose, 16% casein and 4% minerals & vitamins) was prepared at Dairy Science Department, Faculty of Agriculture, Cairo University.

Commercial diagnostic kits for the determination of: serum glucose, activities of aspartate and alanine aminotransferase (ALT&AST), total cholesterol (TC), liver triglycerides, tissue malondialdehyde and tissue glutathione were obtained from Biodiagnostic, Egypt. All other chemicals used in this study were of analytical grade.

## Methods

### Experimental procedure

7 groups of male Wistar rats (5 rats each) were submitted to the following treatments: group 1: negative control (rats were fed on the basal diet), group 2: positive control rats were fed on high carbohydrate fat free diet (HCFFD) to induce fatty liver according to [11], groups 3, 4, 5, 6 and 7; rats were fed on HCFFD and at the same time orally administrated 0.15 g/day/rat of WPI, WPH, α-LA, β -LG and GMP as a suspension in 2 ml water, respectively [23]. After 28 days, animals were sacrificed and blood samples were collected from retro-orbital venus plexus from all animals in plain test tubes.

### Biochemical marker

The activities of serum aspartate and alanine aminotransferase (AST&ALT), serum glucose, total cholesterol (TC), liver triglycerides (TG), glutathione and malondialdehyde were measured using the colorimetric methods described in the kits from Biodiagnostic, Egypt.

### Histological evaluation

After rats were sacrificed, their livers were weighed and liver tissue samples were taken immediately and placed in 10% buffered formalin and subsequently embedded in paraffin. Hepatic index was obtained by dividing liver weight by rat weight × 100. Liver sections were stained with hematoxylin and eosin using standard techniques. Fat accumulation in liver sections was observed as mentioned by [24].

### Statistical analysis

Data are expressed as the mean ± standard deviation (SD). Data were analyzed by one-way analysis of variance (ANOVA), followed by assessment of differences by Tukey's post-hoc test. All statistical calculations were performed using SPSS version 16.0 [25]. Results were considered statistically significant at P < 0.05.

## Competing interests

The authors declare that they have no competing interests.

## Authors' contributions

SHT carried out the study design, participated in data organization, wrote and revised the manuscript; EMH and MMA carried out most of the experiments, participated in data organization, wrote and revised the manuscript; AIA participated in the design of the study and revised the manuscript; MZS carried out the study design and revised the manuscript. All authors read and approved the final manuscript.
